# Current Stimuli-Responsive Mesoporous Silica Nanoparticles for Cancer Therapy

**DOI:** 10.3390/pharmaceutics13010071

**Published:** 2021-01-07

**Authors:** Thashini Moodley, Moganavelli Singh

**Affiliations:** Nano-Gene and Drug Delivery Group, Discipline of Biochemistry, School of Life Sciences, University of Kwa-Zulu Natal, Private Bag X54001, Durban 4000, South Africa; 210506695@stu.ukzn.ac.za

**Keywords:** cancer, nanotechnology, mesoporous silica nanoparticles, stimuli-responsive, targeting

## Abstract

With increasing incidence and mortality rates, cancer remains one of the most devastating global non-communicable diseases. Restricted dosages and decreased bioavailability, often results in lower therapeutic outcomes, triggering the development of resistance to conventionally used drug/gene therapeutics. The development of novel therapeutic strategies using multimodal nanotechnology to enhance specificity, increase bioavailability and biostability of therapeutics with favorable outcomes is critical. Gated vectors that respond to endogenous or exogenous stimuli, and promote targeted tumor delivery without prematurely cargo loss are ideal. Mesoporous silica nanoparticles (MSNs) are effective delivery systems for a variety of therapeutic agents in cancer therapy. MSNs possess a rigid framework and large surface area that can incorporate supramolecular constructs and varying metal species that allow for stimuli-responsive controlled release functions. Its high interior loading capacity can incorporate combination drug/gene therapeutic agents, conferring increased bioavailability and biostability of the therapeutic cargo. Significant advances in the engineering of MSNs structural and physiochemical characteristics have since seen the development of nanodevices with promising in vivo potential. In this review, current trends of multimodal MSNs being developed and their use in stimuli-responsive passive and active targeting in cancer therapy will be discussed, focusing on light, redox, pH, and temperature stimuli.

## 1. Introduction

Cancer is a complex and multifactorial disease characterized by abnormal and uncontrolled cell division resulting in malignant growth or tumors that may spread systematically. With millions of deaths reported annually across the globe, this aggressive and invasive disease is the second leading cause of death both in developing and developed countries. Lung, liver, and colorectal cancers are listed as being the most lethal. Currently, conventional cancer therapy encompasses surgery, chemotherapy and radiotherapy, which can be taxing both physiologically, mentally and financially. Chemotherapy is a systemic-based approach which employs drugs such as alkylating agents (e.g., cyclophosphamide), topoisomerase inhibitors (e.g., camptothecin), anthracyclines (daunorubicin), plant alkaloids (vinblastine) or purine and pyrimidine anti-metabolites (e.g., 5-fluorouracil, mercaptopurine) to target rapidly growing and dividing cells. These chemotherapeutic drugs commonly target dividing cells by blocking key metabolites necessary for replication, by intercalating between DNA, competing with nucleotides, or blocking microtubule formation ultimately leading to cell arrest and cell death [1,2].

Recent decades saw an increase in the screening and development of newly designed drugs with potential anticancer properties. However, about 40% of these newly designed drugs are biomolecules such as peptides, oligonucleotides, proteins and DNA that exhibit low bioavailability and are thus rejected as pharmaceuticals [3,4]. Conventional anticancer drugs commonly used in chemotherapy are usually highly cytotoxic drugs with lowered water solubility, decreased drug stability and bioavailability, and a lack of specificity [5,6]. This often results in severe side effects such as pain [7], nausea [8,9,10], diarrhea, cardiotoxicity, hair loss [11] and depression of the immune system [1]. They are thus used in lowered dosages resulting in lower therapeutic outcomes and may trigger the development of resistance to these therapies [5,12].

Radiation therapy uses ionizing radiation targeted to localized tumor microenvironments and is generally used in conjunction with chemotherapy [13]. It may be applied as neoadjuvant therapy (pre-surgery), adjuvant therapy (following surgery) or concomitant therapy (radiotherapy and chemotherapy used together without surgical intervention) in a multi-faceted approach to prevent cancer metastases and resistance from one single therapeutic approach. Ionizing radiation targets the DNA backbone, causing DNA strand breaks, while also producing reactive oxygen species (ROS) that damage genomic DNA in multiple ways, arresting cellular replication and metabolism [14]. The type of treatment is usually defined by the type of cancer, the stage of cancer, hormone receptivity and lymph node inclusion [13,14,15].

Despite the rapid advances and significant improvements to traditional cancer therapy, there remain several gaping drawbacks, especially the aforementioned side effects associated with the compounds, administration routes, and the overall lack of the specificity of the treatment to the tumor site. Thus, novel approaches must be developed to allow for improved patient care and to complement current traditional therapies. The advent of targeted cancer therapy has shown enhanced clinical efficacy [16,17]. Clinical trials have highlighted the positive outcomes using a combinatorial therapy approach [18,19,20,21,22,23], in which several classes of chemotherapeutic drugs, together with immunotherapy [24,25,26], and/or radiotherapy [27,28], and/or hormone therapy [29,30,31,32,33], are used. A disadvantage of this therapy is that it often results in a decrease in the patients’ physiological and psychological care [8,10].

Consequently, there is an overwhelming niche for tailored delivery systems that can improve the biostability, bioavailability and cost-effectiveness of existing developed compounds and molecules [34]. These delivery systems should also be edited to combine several therapies for a single, multi-faceted approach that will have the potential to minimize drug resistance and cancer recurrence [35].

## 2. Controlled Gene and Drug Delivery Systems

Through the introduction of combinatorial chemistry and high-throughput screening, a variety of active pharmaceutical agents have been produced and tested extensively for their anticancer activity. However, a large number of these drugs are lipophilic and poorly water-soluble, contributing to their poor bioavailability and inadequate dissolution rates in the gastrointestinal tract [36]. The safe delivery of therapeutics to the human body is imperative to the effectiveness of the agent being used, and its overall efficacy in the desired target tissue. There has been a steady increase in the development and enhancement of delivery strategies, with interest in the conjugation of a functional biomacromolecule partner [37,38,39]. This may then be administered by either oral or parenteral routes, with the latter including intravenous, intraperitoneal or intramuscular methods [40]. There are two notable serum concentration limits: a lower limit that shows the minimal concentration at which a drug can effectuate a therapeutic response, and an upper limit at which the drug becomes toxic and may elicit a harmful reaction [41,42].

**Figure 1:**
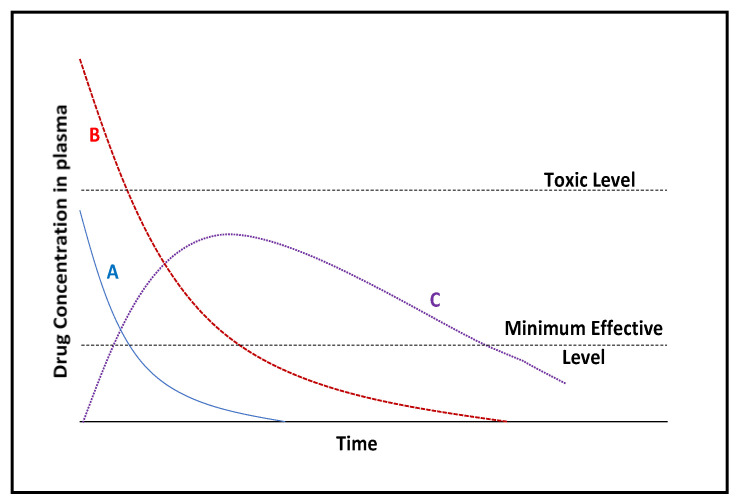
Drug serum concentration versus time for conventional dosage forms and an ideal slow-release dosage. Curve A (blue): Intravenous administration; B (red): intramuscular; C (purple): slow-release administration [adapted from 43]. A slow-release administration route allows for a drug serum concentration that is effective, non-toxic and sustained over a longer period in comparison to conventional administration routes.

Ideally, a controlled release dosage that can maintain a therapeutic concentration of the drug in serum throughout the dosing interval is illustrated by curve C in Figure 1. With this in mind, the development of delivery vehicles that can maintain a sustained release of a drug, with negligible interaction with the drug and maintain biocompatibility of the complex is desired [43].

These formulations aim to extend the serum half-life of these drugs and maintain zero-order release kinetics over an extended period. These preparations must also overcome temperature, pH or mechanical stresses and susceptibility to degradation by esterases [44]. There is a growing need for complementary strategies to overcome these challenges by contributing to the development of a biocompatible delivery vehicle that can load a saturated drug suspension and effectively release a sustained and predictable therapeutic concentration of the drug.

### 2.1. MSNs in Nanotherapeutics

**Figure 2:**
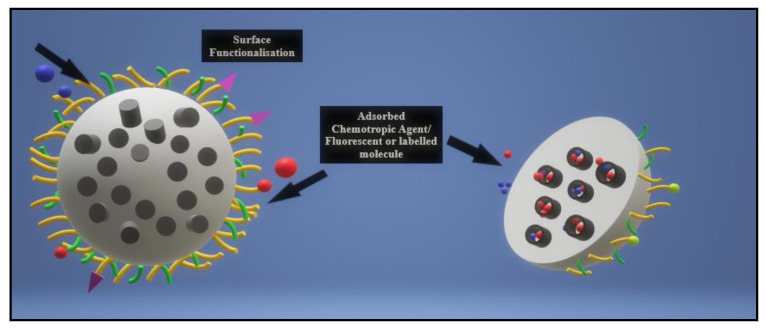
Illustration of a functionalization of a mesoporous silica nanoparticle. MSNs may be functionalized on the outer surface with targeting ligands or polymers for enhanced biocompatibility, while the inner pores may be loaded with chemotherapeutic agents, drugs, genetic components or fluorescent/labeled molecules for easy imaging.

Since the advent of periodically ordered mesoporous silica by the research company Mobil in 1992, the organic polymeric-inorganic core-shell hybrid nanoparticle viz. mesoporous silica nanoparticle (MSN), pictured below in Figure 2 has gained attention for its application as a safe, efficient, and multifunctional drug delivery vehicle [45,46].

MSNs, have rapidly become important candidates in nanomedical applications since a MCM-41-type mesoporous silica material was first reported as a drug delivery system in 2001 [47]. MSNs can be described as having a unique mesoporous structure encompassing a solid framework with a porous honeycomb-like structure, and a large active outer and inner surface area, allowing for the attachment of different functional groups for cell-specific targeting of the drug moiety. Their structure is riddled with hundreds of empty channels (mesopores) that can absorb or encapsulate relatively large amounts of bioactive molecules. Their properties, such as tunable particle size (range of 50–300 nm), no significant cytotoxicity, high surface area, two functional surface areas, large pore volume, tunable pore size with a uniform and narrow distribution (2–10 nm), and good chemical and thermal stability due to their rigid framework, make MSNs suitable for various controlled release applications [48,49].

The most promising advantage of MSNs as a drug delivery system is their “zero premature controlled release” property [49]. Within their honeycomb-like 2D hexagonal porous structure are cylindrical pores that run from end to end, with no interconnectivity between the porous channels, allowing drugs to be delivered precisely without leaching before reaching the targeted cells or tissues. This “no leaking” function is present even in the case of incomplete capping of the outer surface, suggesting that the individual cylindrical pore channels act as independent reservoirs for drug adsorption and release [47,50]. MSNs possess advantages over traditional nano-based formulations, especially for cancer therapy. Their low toxicity, high drug loading capacity and comparatively better biocompatibility than other metal oxides has increased their desirability as drug delivery vehicles [51,52,53].

Enhanced development procedures for MSNs, including structure design [54,55,56,57], biosafety profile characterization [58,59], biodistribution [51,57,60,61], and mechanisms of excretion studies [60,62], have been reported. MSN’s multi-functionality and enumerable capabilities have seen them being used in bioimaging for diagnostics (fluorescence imaging or magnetic resonance imaging) [35,51,63,64], biosensing and as biocatalysts [65], bone repair [66], scaffold engineering [66,67,68,69,70], therapeutic devices (drug delivery [45,49,50,71,72,73,74,75] or photothermal therapy [63,76]), and as theragnostic agents [54] (single nanocarriers that are capable of combining the diagnostic and therapeutic functions) [47,73,77].

One of the advantages of MSNs is their ability to be manipulated and modified to enhance biocompatibility, cellular uptake and binding affinity [54]. The abundant silanol groups on the MSN surface can be actively functionalized to increase positive or negative charges, correct for hydrophobicity or hydrophilicity, improve targeting functions or to support controlled drug release [55,57] (Table 1). These modifications can be accomplished by organosiloxane or siloxane co-condensation, post-synthesis grafting and molecular imprinting [54].

This led to the optimization of MSNs’ size, architecture, and surface properties to allow for the addition of stealth agents and/or targeting ligands to enhance biocompatibility, biodistribution and accumulation at the tumor site [57,78]. Furthermore, tracking agents such as quantum dots, iron oxide NPs or fluorescent dyes have also been incorporated into MSNs for monitoring of the NP’s fate in the human body [64].

### 2.2. Stimuli-Triggered MSNs

Cancerous tissue distinctly possesses leaky vasculature and epithelium with abnormal fenestrations varying between 400 nm. Increased accumulation of apt-sized NPs in cancerous tissue occurs through the enhanced permeability and retention (EPR) effect, which is directed by rapid tumor growth and the subsequent formation of new blood vessels or angiogenesis. Thus, NPs designed for passive tumor targeting are subject to size restrictions and functionalization of the outer matrix to allow for favorable cellular uptake. Additionally, the tumor microenvironment demonstrates a significant difference from that of normal functioning cells, and have hence become a targetable deviation which NPs should differentiate between [79].

The tumor microenvironment and its abnormal epithelium serve as a functioning system which creates a constant inflammatory state, incapable of repair, with a marked dysfunctional shape, size and cellular/bioenergetic metabolism patterns. The resultant tumor microenvironment is hypoxic (relies on aerobic glycolysis) and acidic. This is due to the rapid tumor growth, and the diffusion of oxygen and nutrients is limited. Metabolites such as lactate and glutathione, then accumulate, resulting in the tumor core becoming necrotic, surrounded by a peri-necrotic hypoxic cloistering of abnormal dividing cells [80]. The exploitation of metabolic targets can be achieved through direct means (metabolic enzymes) or indirect targets of disordered signaling pathways and the resultant microenvironment created.

**Figure 3:**
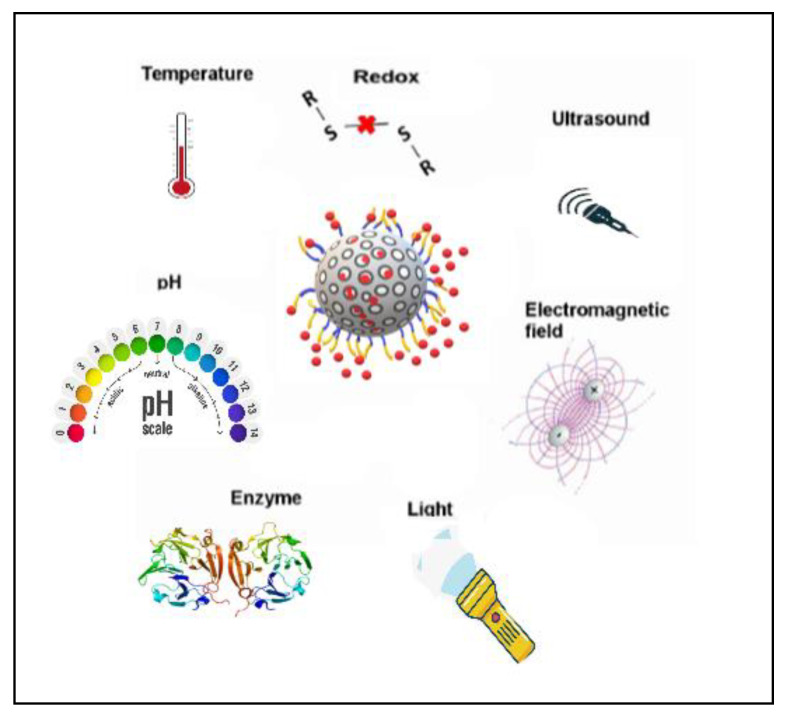
Illustration of various stimuli-gatekeeping mechanisms being developed for mesoporous silica nanoparticles.

MSNs can be designed as a stimuli-responsive trigger system for the controlled release of a drug. Several regulating mechanisms have been designed, and their feasibility and effectiveness evaluated. The triggers chosen may be internally located and responsive within a specific environment, or externally activated by non-invasive means such as light, pH, redox potential, temperature and enzymes (Figure 3) [81].

Incomplete capping involves the use of polymerized or lipid bilayers surrounding the external surface, which undergoes competitive displacement when triggered by a stimulus, allowing a slow release of cargo into the desired tumor site. Generally, two gating strategies have been used, one of which relies on the use of a mesopore-sized macromolecule that can block the pore opening and respond to an applied stimulus that induces a conformational change. Examples include the attachment of gold nanoparticles [82], CdS [83] and Fe_3_O_4_ [84] nanoparticles to the surface of MSN to block the pore openings unless stimulated by photothermal, redox, pH or enzyme-sensitive stimuli.

Alternatively, the MSN can be coated with a polymeric covering that undergoes degradation when exposed to a stimulus. Polymeric micelles, including MSNs that have been coated with cross-linking organic and/or inorganic polymers or lipid bilayers that enclose drug that has been electrostatically loaded onto the MSN core. Moodley et al., 2020 described a polyelectrolyte coated MSN that combined organic and inorganic co-polymers grafted on to the superficial surface of the MSN creating a brush-like covering that showed pH-sensitive release of loaded drug [85].

In addition to the advanced development of many novel trigger-response systems, there has been the evolution of dual [37,77,86,87] or tri-stimuli-responsive [53,86,88] systems that include pH and redox-responsive systems, thermal and pH-responsive systems and recently a pH, reduction and light triple-responsive system using MSNs [89]. However, with these dual functioning stimuli-responsive systems, it is necessary to account for the preparation time, cost and feasibility of the final particle, especially for clinical or commercial applications [53].

### 2.3. Phototherapeutic MSNs

**Figure 4:**
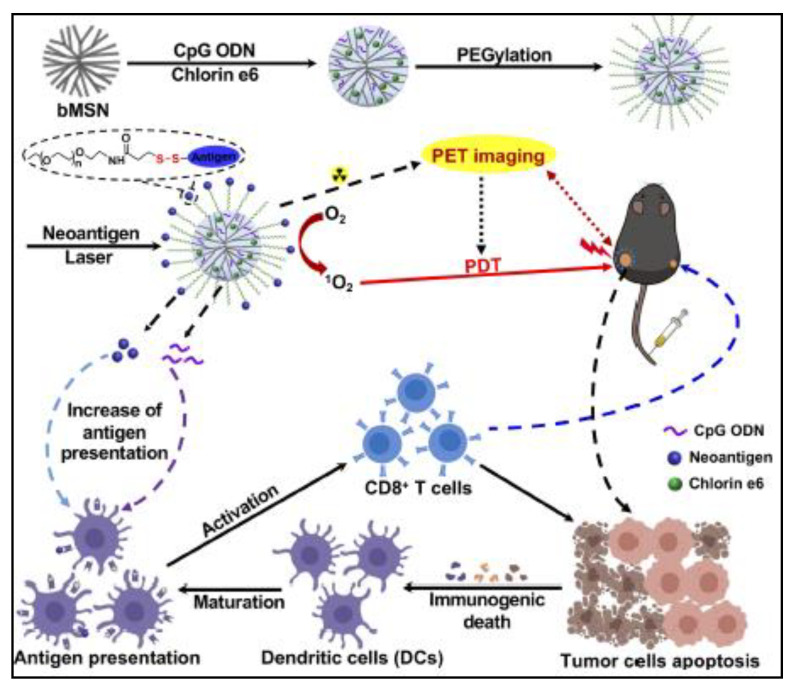
Schematic illustration of PET-guided PDT MSN used by Xu et al., 2019 [90]. Reprinted with permission from Xu et al., 2019. Copyright (2019) American Chemical Society.

Conventional photothermal therapy (PTT) uses the conversion of photonic energy to heat to thermally ablate cancerous cells. Photothermal therapy can be applied specifically and can be spatiotemporally controlled. The application of a light stimulus can be achieved for a defined duration and area. The light used is usually near-infrared radiation which is relatively non-invasive. Xu et al., 2019 devised a theranostic MSN co-loaded with neoantigen peptides, CpG oligodeoxynucleotide adjuvant, and the photosensitizer chlorin e6, which post-administration in murine bilateral tumors subsequently underwent laser irradiation (Figure 4). Using Positron Emission Tomography guided photodynamic immunotherapy, dendritic cells and neoantigen-specific cytotoxic T-cell lymphocytes were raised at treated tumor sites resulting in significant anti-tumor activity [90]. Immunotherapy stimulated treatments are highly regarded as they rely on patient-specific gene sequencing to effect change in targeted tumor tissue. This has potential for cancer-specific vaccination development using stimuli-responsive systems.

Commonly defined light-responsive systems use gold nanoparticles that are adhered to the surface of MSN pores using a linker. The cargo release is triggered by photo dimerization or the photo-cleavage of the linker polymer due to the photothermal energy created by irradiated gold nanoparticles. A wide array of photo-labile polymers and compounds have been investigated for their biocompatibility as a photo-inducible gating compound for biomedical applications. UV light (~250 nm) has been used as a trigger for reversible photo-responsive systems, as light at this wavelength causes photo dimerization of known coumarin, the transformation of azobenzene moieties and photoisomerization of spiropyrans.

Within the visible light spectrum, light within the wavelength range of 650–950 nm displays deeper tissue penetration with minimal phototoxicity and is highly desirable for photo-responsive therapy. Li et al., 2020 devised a red-light responsive MSN system that used a heptamine cyanine dye that was attached to the surface of MSNs-doped with DOX, which was further encapsulated by PEG. Light (700 nm) triggered the photooxidative cleavage of the photolabile linker, releasing the encapsulated DOX in xenografted 4T1 tumor-bearing BALB/c mice [91].

Salinas and colleagues (2020) used two photo-sensitive ruthenium complexes to gate MSNs loaded with safranin O. Ruthenium complexes as gatekeeping molecules for MSN systems have been investigated previously in cancer treatment, and in this study the complexes underwent photo substitution of their pyridine ligands by solvent molecules under visible light, releasing the loaded dye. This was reversed when exposed to a temperature of 80 ℃, closing the pores and rebinding the ruthenium complexes to MSN [92].

Using triphenylphosphine modified MSN loaded with DOX and indocyanine green (ICG) via the linker L-menthol, Shi et al., 2019 were able to target the mitochondria of cancer cells directly. NIR-laser radiation-induced ICG-mediated photothermal anticancer activity, and simultaneously induced the photo-transformation of l-menthol, releasing DOX into cancer cells [93].

### 2.4. Redox-Triggered MSNs

Glutathione (GSH) in a cysteine-containing tripeptide that is a key regulating element of intracellular redox conditions and other cell-cycle related processes. It functions in reducing protein disulphides, detoxifying free radicals and exogenous toxins, and maintaining the intracellular redox balance through its heterogeneous forms. GSH levels vary significantly between the exterior (2 μM) and interior (10 mM) of cells. Within the tumor microenvironment, significant heterogeneity of redox conditions is displayed, with GSH levels being monitored to be at least 4-fold higher in concentration than in normal tissue.

Nanodevice systems designed for redox sensitivity react specifically to endogenous intracellular signals specific to the tumor microenvironment. Standard redox-sensitive systems developed to involve the use of disulphide linkages which can be cleaved by GSH upon entry into cancer cells and selectively trigger the release of the cargo into the targeted cancer cell.

An earlier redox-responsive MSN system was developed based on the conjugation of polyethylene glycol (PEG) to the surface of MSNs via a disulphide bond. The release of drug was triggered by the addition of glutathione, or a reductive environment. Gatekeepers used in redox-responsive systems include polymers [94], polypeptides [95], hyaluronic acids [73] and β-CD [96]. One such model developed by Chen et al., 2020 grafted the tumor specific anti-CAIX antibody via disulphide linkages which dissolved under GSH exposure at tumor sites, releasing the loaded DOX into mouse breast cancer cells [97].

Another redox-responsive system described by Liu et al., 2020 used β-cyclodextrin (β-CD) as a gatekeeping molecule grafted to the surface of MSN via a ferrocene-containing PEG-b-PMAFc (PPFc) co-polymer. DOX was loaded into the core of the MSN and the cross-linked co-polymer conjugated to β-CD, providing a polymeric coating trapping the drug until exposure to oxidative stress [98]. Cui et al., 2019 described a redox-sensitive hybrid system that contained a MSN core doped with fluorescein dye, covered with a disulphide linked PEG outer coating of 10–30 nm in thickness. Upon reaction with higher GSH levels, cleavage of the disulphide bonds occurred, releasing the PEG outer shell and expelling the drug from the MSN matrix [99].

In a similar hybrid formulation, a GSH-sensitive disulphide linked MSN was further coated with a pseudo-tripeptide of cystine-dopamine (Cy-DA) encapsulating DOX and rhodamine B which was able to respond to both oxidative stresses and exposure from the enzyme pepsin. This was of particular interest to stomach-related diseases and cancer therapy [100].

### 2.5. pH-Responsive MSNs

From the many endogenous stimuli-triggered MSNs being investigated, pH stimuli-responsive MSNs remains one of the more popular designs due to the relative reliability of pH-sensitive moieties that are grafted to the surface of MSNs. They also stimulate the permeation of the internalized cargo through hydrophobic/hydrophilic transformation of the immobilized molecules gating the surface of MSNs’ pores The pH triggered linkers use the varying pH microenvironments within the cell (pH ~5.6), the suggested acidic environment of the tumor or inflammatory sites (pH ~6.8) and the stomach (pH 1.5–3.5). They are usually inert at physiological pH (7.4) and respond to a drop in the pH environment. Most pH-responsive systems use pH-labile chemical bonds that can be grafted onto the surface or coated on the surface of the nanoparticle [53,71,101]. Hydrolyzable bonds such as amide, imine, acetal, ketal, ester and hydrazine linkers or ionizable polymer/lipid bilayers which rely on electrostatic interaction with surrounding media, are used to selectively cap the porous surface of the MSNs.

The earliest reported pH-responsive system was an anion triggered drug delivery system, which when faced with high pH values, triggered “open” state releasing squaraine and vitamin B2 [102]. Another approach using pH triggers involved using gatekeeping molecules such as poly-l-histidine which have p*K*a values near the interstitial tumor pH. Small fluctuations in the pH cause protonation of the multiple functional groups and change the overall solubility of the gatekeeping molecule [103]. MSNs coated with gatekeeping molecules that block release by electrostatic interactions include the use of a polyelectrolyte multilayer (PEM) that is coated on the MSN by layer-by-layer technology. PEMs can consist of chitosan (CHIT) [104], alginate [59], sodium poly(styrene sulfonate) [105] and dialdehyde starch [106]. The use of chitosan is highly advantageous as it is not limited in availability, is recyclable, does not require preparation and is biocompatible and biodegradable [107,108,109]. CHIT dissolves at acidic pH, which is preferable for acidic-responsive release systems, and the –OH and –NH_2_ groups can be modified for dual functionality [103].

A polyelectrolyte coated MSN that showed pH-responsive drug release of 5-fluorouracil (5-FU) and doxorubicin, respectively in a cancer cell models was recently reported. It was concluded that the polymeric brush-coating of TPP, chitosan and PEG when protonated underwent a transformation, opening the MSN pores and releasing the drugs into the targeted cancer cells [85,110]. Yan et al., 2019 developed a MSN system capped with chitosan and folic acid for the pH-responsive release of both a chemotropic drug, DOX and a photosensitizer (pheophorbide a, PA) for targeted and synergistic cancer therapy [111]. Peng et al., 2019 designed a MSN coated with a pH-sensitive linker that attached a Schiff base co-polymer layer, which enclosed the chemotropic drug doxorubicin. The imine-bond linker dissolved at lower pH, releasing a significantly larger amount of drug than at physiological pH [112]. Ryplida et al., 2019 devised a pH-sensitive MSN with a core containing carbonized zwitterionic PEG-grafted poly[(dimethylamino)ethyl methacrylate-co-sulfobetaine methacrylate] (PEG-g-PDS) and the photothermal dye indocyanine green (ICG). As this carbon dot (CD)-MSN hybrid complex was taken in by cancer cells, the ICG-complexed to CDs generated sufficient photothermal heat to kill the cancer cells at an acidic pH [83].

The pH-responsive gating systems developed are often prone to lowered loading and release efficiencies, particularly depending on the electrostatic effect produced by the gatekeeping polymer and its interaction with the superficial surface of the MSN. Because of the unpredictability of grafting biopolymers to the MSN surface, the distribution and toxicity of gatekeeping polymers vary accordingly. This ultimately may affect the loaded drug’s performance. There is also a propensity of leaching the loaded bioactive drug before it reached targeted sites. Aggregation of the polymer and the possible pharmacokinetic implications of complicated gating systems hinders the translation of developing gated-response systems. Thus, there is a growing interest in simplistic designed MSN gated systems, including the use of end-capped MSNs, which incorporate peptides or gating mechanisms into the MSN core through co-condensation.

Zhao et al., 2019 presented a pH-responsive-end-capped MSN that incorporated a biofunctional hybrid peptide (P45) containing the RGD peptide linked to the N-terminal domain of P41, based on the C-terminal domain of human matrilin-1 which enclosed the chemotropic drug DOX inside the porous core [113]. Upon protonation upon entry into an acidic environment, the peptide gatekeeper disassembled and opened the pores, allowing the release of DOX from the MSN matrix. Additionally, the monitored fluorescent release of DOX showed significant release in A549 cells, weakly in MCF-7 cells and negligibly in HEK293 T cells, highlighting the specificity of this particle for cancer treatment.

### 2.6. Temperature-Sensitive MSNs

The design of temperature-responsive MSN delivery systems relies on the use of thermosensitive polymers as gatekeeping molecules, which undergo a temperature-dependent phase transition. This is particularly of use in tumor tissue with heightened inflammatory markers that show a significant temperature variation in which thermosensitive MSN delivery systems can be employed beneficially.

Yu et al., 2017 designed a ROS-responsive MSN using the temperature-sensitive 4-(4,4,5,5-tetramethyl-1,3,2-dioxaborolan-2-yl) benzyl acrylate modified polymers (ROSP) to selectively gate DOX inside MSN below the lower critical solution temperature (LCST) with zero premature release until an external or endogenous oxidative stress was applied. The loaded drug was released in response to oxidative stress, which induced oxidation of the hydrophobic groups in the co-polymer resulting in an increase in temperature to above physiological temperature (37 ℃). The gating co-polymer then transformed to release the drug to the targeted cancer cells [114].

The thermosensitive polymer poly (N-isopropyl acrylamide (PNIPAAm) and its derivative have been used as temperature-sensitive gating molecules. They undergo hydrophobic transformation to a collapsed form during exposure to higher temperatures (above LCST, ~50 ℃). Amgoth et al., 2017 synthesized a MSN delivery system with a combination of [(PNIPAM)-b-(Glycine)] tethered to the superficial surface of MSN, gating the drug imatinib mesylate. They successfully inhibited the growth of leukemia cancer (K562) cells after a 24-h exposure [115]. Cui et al., 2019 used the temperature-sensitive synthetic polypeptide poly (γ-benzyl-L-glutamate) (PBLG) derivative to selectively gate DOX by forming a brush-like coating around the porous silicon matrix. The PBLG polymer was further modified to incorporate disulphide bonds and finally folic acid for targeted cancer-specific delivery. Thus, this system was responsive to both increasing GSH levels, through the dissolution of its disulphide bond and upon exposure to higher temperatures, the co-polymer underwent a conformational change opening up the pores for release of the drug into cancer cells [99].

### 2.7. Multi-Stimuli MSNs

MSNs large surface area and interior capacity coupled with its tunable pore volume and size provide an open canvas for the engineering of novel multi- application nanotherapeutic devices. Of interest is the use of organic and inorganic nanomaterials that can be tethered to the surface MSN and impart optical, chemical, electronic, magnetic or physical transformations to the MSN matrix, improving their overall bioavailability and pharmacokinetics in vivo. For enhanced effectiveness, the combination of multifunctional ligands enhances the use of MSNs as theranostic devices capable of synergistic nanoscale functionality. This has seen the increased use of various carbohydrates, proteins, lipids and inorganic compounds to improve particle biostability, effectiveness and controlled release behavior. Multi-stimuli-responsive nano-systems being developed aim to enhance the anticancer activity, increase cancer specificity, lower bio-toxicity and provide a control element for targeted delivery based on the tumor architecture. Theranostic MSN systems can introduce a molecular probe, tracking dye or contrast agent that allows for imaging-guided therapy development.

Some highlighted multi-stimuli systems that are currently being investigated include a multi-responsive MSN system that was coated with polydopamine via a disulphide linker to a DOX-loaded MSN, which showed photothermal, redox and pH sensitivity [116]. In a multi-stimuli-responsive system, the delivery of DOX was realized using mesoporous silica encapsulating a mesoporous carbon nanoparticle, which was further coated with carbon dots using a disulphide linker. As the nanoparticle was internalized, interior GSH levels caused protonation of the disulphide linker, releasing the carbon dots for easy fluorescent detection. DOX was further released for its chemotropic effect, which coupled with irradiation-induced photothermal effects and created synergistic inhibition of 4T1 mouse breast cancer cells [117].

The innovative design of stimuli-responsive delivery vehicles has emerged as a promising tool against conventional chemotherapeutic approaches due to its control-release functions and improved biotoxicity. Further investigation into the possible pharmacokinetic implications in vivo is required for translation of these nanodevices in clinical trials. However, the good biosafety, bioavailability and multi-functionality enhance their relevance and likely clinical advancement in cancer therapeutics.

Table 2 highlights some of the emerging multifunctional uses of MSNs as biomedical nanodevices in various theranostic applications within the last two years.

## 3. Summary, Conclusion, and Future Perpectives

There is a significant amount of pre-clinical data available elucidating the use of light, redox, pH, temperature, and various stimuli-responsive NP systems developed, highlighting their promising efficacy and pharmacokinetic outcomes in in vivo models. However, the foremost impediment to clinical translation of MSN systems developed remains the discriminatory delivery of drugs/genes systemically to target tissues. Table 3 provides a summary of the trends in stimuli-responsive MSNs from 2018–2020. There are multiple pathways that internalize MSNs including passive routes such as phagocytosis and pinocytosis which are further affected by the size, charge and functionality of the designed NP. Additionally, the rate, quantity and localization of the internalized NP is subject to the design of the nanodevice and its responsiveness to intracellular signals. MSNs in particular, may foremost be affected by the tubular pore design of its cargo “reservoir” and the size of the opened pore in vivo. Thereafter, the gating mechanism can be accomplished by either sealing the pore using similar sized gating molecules, or by coating of the gating bilayer which results in the release of cargo by conformational transformation of the gating linker or disintegration of the gating layer.

With systemic delivery, fluctuations of endogenous signals vary significantly through the different tissues and within the subcellular compartments of the cell. In tumor cells, the dissimilarity between regulated metabolic levels and cellular activity becomes more distinct. Stimuli-responsive systems that respond to endogenous signals such as pH, redox or slight temperature changes are further faced with the challenge of the effectiveness of their gating linker to environmentally stimulated conformational transformations, slight fluctuations in metabolic levels that may cause premature release of the cargo, unwanted electrostatic interaction with gating linkers still adhered to the interfacial surface of MSN, or exceedingly small bioactive molecules being trapped in the pores by larger molecule linkers due to negligible electrostatic interaction during cargo release. Therefore, the design of these stimuli-release MSNs needs to be optimized for enhanced biostability and effectiveness in vivo to prevent unwanted accumulation of the NP in the non-target tissues. The loading and release of electrostatically sensitive drug/gene components needs to be better developed to prevent low loading and release efficiencies seen in these stimuli systems. Nevertheless, due to the relative abundance of electrostatically pliable material available, pH-responsive MSNs remain the most investigated stimuli-sensitive device being developed today.

Exogenous stimuli-responsive systems such as the use of photo-sensitive polymer decorated MSNs in photothermal therapy (Table 3) or dye-doped MSNs in image guided therapy is an attractive field for nanotechnology development in biomedicine, due to the instantaneous results that are obtained by the application of a controlled stimulus. The first silica nanoparticle mentioned with clinical translation was a dye-doped MSN used for real-time imaging of cancer sites, which is still being applied in nodal cancers for long-term testing. There are two clinical trials of photothermal responsive silica NPs currently undergoing recruitment, and two trials with published results. These silica NPs were tested over a one-year duration and lack long-term validity; however the use of photo-sensitive silica NPs holds promise in cancer and atherosclerosis treatment as application of the light stimulus is directed at the targeted site and negates any unwanted adverse systemic effects [131,132].

Multi-stimuli-responsive MSNs typically combine two or more stimuli-responsive components for conjugation onto the superficial surface of the MSN. This allows for enhanced targeted uptake, and the potential decrease of premature leakage at undesirable sites. Current systems have shown encouraging pre-clinical results reducing biotoxicity by targeting multiple features specific only to the tumor microenvironment. Additionally, theranostic MSNs promise the delivery of bioactive molecules capable of being tracked by real-time imaging simultaneously with the delivery of chemotropic agents. This may reduce unnecessary scans by providing real-time monitoring and reduce the need for conventionally applied radiation prior to removal of tumors. Extensive pharmacokinetic fate and in vivo testing is warranted before further clinical translation can be undertaken.

MSN delivery may face adverse swelling effects, slight diffusion of internalized cargo prior to entry into the targeted site, slow degradation rates of gating material and MSN matrix dissolution. The ultimate pharmacokinetic outcome of the MSN, gating material and delivered bioactive molecule or gene component is then affected by the optimization of the MSN design, use of linker, the linkers’ transformational effects in vivo, response to a desired stimulus, the potency of the response, the magnitude and recurrence of an applied stimuli necessary to effectuate a response and the effect of the applied stimulus on surrounding healthy tissue. This needs to be thoroughly investigated pre-clinically as mentioned in this review, before further translation can be seen. With the growing trend of research and clinical adaptation of silica nanodevices, stimuli-responsive MSNs is a propitious advancement toward a solution to a variety of existing diseases and warrants future development. Their good biosafety, bioavailability and multi-functionality enhance their relevance and likely clinical advancement in cancer therapeutics.

## Figures and Tables

**Table 1:** Common methods to attach functional groups onto MSNs surface.

Functional Groups	Common Surface Functionalization Methods
Ureidoalkyl	Co-condensation
Mercaptoalkyl	Co-condensation, grafting
Cyanoalkyl	Co-condensation, grafting
Aminoalkyl	Co-condensation, grafting
Allyl	Co-condensation, grafting
Isicyanatoalkyl	Grafting
Epoxyalkyl	Grafting
Phosphonatoalkyl	Grafting

**Table 2:** Stimuli-responsive MSN systems developed in 2019–2020.

Source of Stimuli	MSN -Formulation	Loaded Drug/Dye	Concluding Remarks
pH	Pt/COOH-MSN [118]	Cisplatin	Two-fold drug release at pH 4.0. Enhanced anticancer activity was noted in A549, A2780 and MCF-7 cells (>84%).
Magnetic	Iron oxide-MSN-FA [119]	gemcitabine HCl	Sustained drug release observed. Anticancer activity in PANC-1 cells
Enzyme (matrix metalloproteinase-2 (MMP-2))	Cisplatin-collagen coated MSN [120]	Cisplatin	Collagen capped MSN released Cis in response to MMP-2 exposure. The anticancer activity was enhanced in A549 cells
Redox	4-(phenylazo)benzoic acid-β-cyclodextrin (β-CD, SNAC)-MSN [121]	DOX	Hypoxic exposure stimulated DOX release
Redox	FITC-CPMA/PEG-CPMA MSN [122]	DOX	Polymeric layering acted as a redox-sensitive coating releasing DOX in treated HeLa cells
Redox, enzyme (MMP)	PLGA/MSNs-PMS [123]	celecoxib and bone growth factor (BMP-2)	GSH and MMP exposure triggered the release of the anti-inflammatory drug and BMP-2
Redox, Light, Enzyme (Hyaluronidase)	CuS-BMSN-HA [124]	DOX	Upon exposure to NIR, there was synergistic photothermal/DOX anticancer activity. Increased targetability by CD44 receptor on HeLa cells
Glucose, pH, redox (GSH)	MSNP-CYS-5FU-FABA@DOX-CD [125]	DOX, 5-Fluoro-2′-deoxyuridine	Anticancer activity was obtained with a dual chemotropic drug formulation against an aggressive murine lymphoma model.
Theranostic	FlexLP-functionalized MSN [126]	Fluorophore	environmental responsive fluorescent probe designed to investigate hydrogen bonding environments. This will allow visualization of subcellular compartments in the tumor microenvironment
pH, redox	Polydopamine-MnO_2_ -albumin–folic acid-MSN [80]	DOX	Tumor-responsive drug release observed in SMMC-7721 cells
pH, redox	MSN-S-S-Chitosan [127]	salicylic acid	Upon exposure to GSH and acidic pH, drug release was enhanced.

**Table 3:** Comparison of trends seen in stimuli-responsive MSNs.

Systems	Photo-Responsive	Redox-Responsive	pH-Responsive	Temperature- Sensitive	Multi-Stimuli-Responsive
Key features of system	Used as a spatio-temporal control function for cargo release. Allows for photo-guided dual therapy or simultaneous photodynamic therapy (increases ROS and photocoagulation)	Responds to raised intracellular levels of reactive oxygen species or redox fluctuations	Responsive to acidic-basic shifts, typically with the use of gating molecules that undergo conformational change with protonation.	Sensitive to change in temperature from LCST to body temperature. MSNs designed may be used for thermal ablation of cancer cells/plaque.	Designed to respond to two or more stimuli that may be either endogenous, exogenous or a combination of both. Typical linkers may use cancer cell features to enhance internalization.
Types of gating material used	Azobenzene derivatives, spiropyrans,	Thioketal species, double disulfide linker molecules	Inorganic polymers- Schiff base sensitive linkers, organic polymers- chitosan	Inorganic polymers such as PBLG or polyurethane derivatives	Conjugated polymers e.g., double disulfide linker joined to the organic chitosan.
Mentions in the literature: 2018–2020 *	GScholar: 1240 Elsevier: 181 MDPI: 22	GScholar:13,000 Elsevier: 203 MDPI: 2	GScholar: 17,100 Elsevier: 1219 MDPI: 44	GScholar:16,300 Elsevier: 1602 MDPI: 16	GScholar:14,500 Elsevier: 483 MDPI: 2
Clinical application and outcomes	2 trials recruiting 2 trial completed: Silica-gold NPs/silica-gold iron NP activated by NIR radiation saw significant regression of treated atherosclerosis. Trial 2 used sNPs for real-time imaging of nodal metastases. [128,129] 1 trial terminated Clinical trial data shows promising results; however there is a need for long-term development and prolonged testing.	-	-	-	1 trial completed: magnetic guided silica-gold NP treated atherosclerosis [130]. 1 trial terminated Long-term studies required for continuance of results obtained. However, results show developed NPs performed better than conventional treatment options giving patients a better quality of life.
Notable features that require development	Light radiation used may either have adverse effects, or have difficulty in penetrating deep layers of tissue.	Redox concentrations are subject to fluctuation, especially when targeting subcellular components. Accumulation may be seen in non-targeted tissue such as lungs, liver and spleen.	Low loading efficiency of drugs and genes. Difficulty in controlling the loading and release of bioactive molecules, as electrostatic effects are continually fluctuating. Accumulation may be seen in non-targeted tissue such as lungs, liver and spleen.	Temperature fluctuates within tissues and is also affected by environmental factors. May require an applied stimulus. Requires gating material with high thermal stability. Thermal ablation may affect changes in surrounding tissue. Further translation is required.	Although developed systems combine responsiveness to applied stimuli, there is also the possibility of a combination of barriers that may affect the NPs performance in vivo.

* Google Scholar: 2018–2020 All results, Elsevier: Research articles only (Mentions in article, abstract, or keywords), MDPI: All results.
